# Supplementary figures and images for: The usefulness of change in CT score for evaluating the activity of Mycobacterium abscessus (Mab) pulmonary disease (Mab-PD)
Source: PLoS One. 2023 Feb 8;18(2):e0281103. doi: 10.1371/journal.pone.0281103 (PMC9907801; doi:10.1371/journal.pone.0281103)

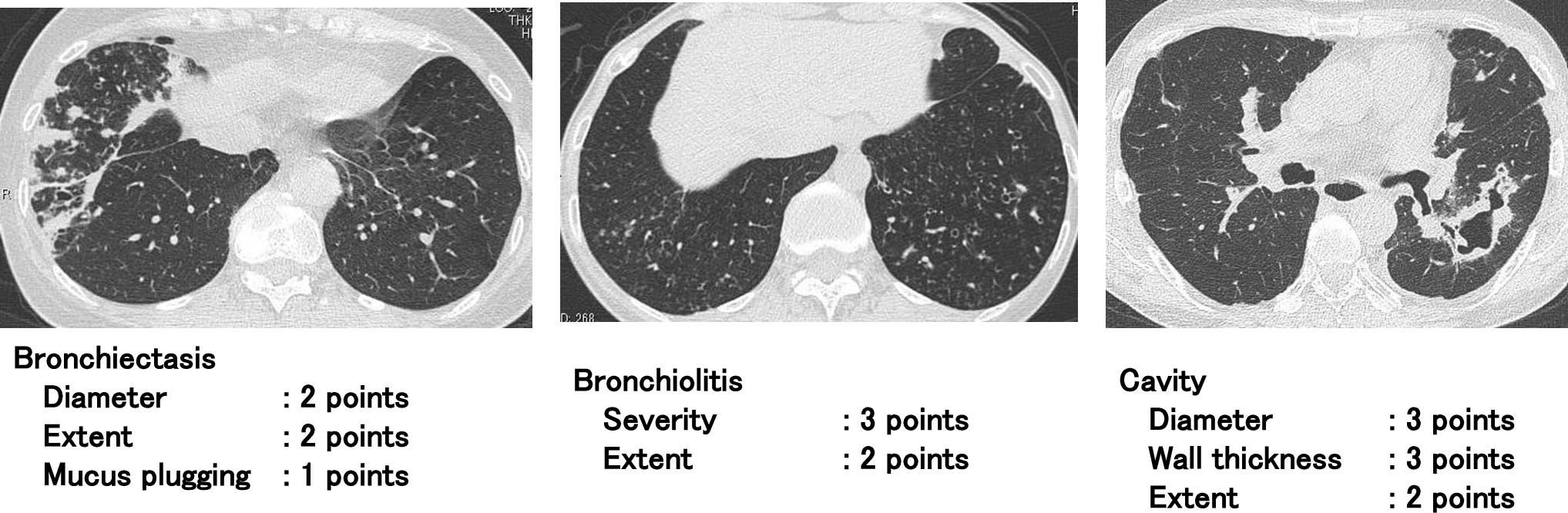

Supplement: S1 Fig — (TIF) [file pone.0281103.s001.tif]
