# Supplementary material for: The usefulness of change in CT score for evaluating the activity of Mycobacterium abscessus (Mab) pulmonary disease (Mab-PD)
Source: PLoS One. 2023 Feb 8;18(2):e0281103. doi: 10.1371/journal.pone.0281103 (PMC9907801; doi:10.1371/journal.pone.0281103)
Supplement: Attachment — Submitted filename: PlosOne Mabs review.rtf [file pone.0281103.s002.rtf]

Reviewer's response
The manuscript written by Hara H et al investigates the usefulness of CT score change in characterizing M. abscessus-associated pulmonary disease in human patients. M. abscessus lung infections represent a significant medical burden and a better measurement of Mabs-PD is needed to improve clinical diagnosis and treatment decisions. Lung CT scores alone remain semi-quantitative. The investigators of this short report analyzed retrospective data obtained on a limited patient cohort (n=32). CT changes could be measured over a 4-year period of time in case of 18 patients and found significant correlations between CT score changes and microbiological or immunological measures of the disease. While the manuscript raises an important question and explores an interesting idea, the presented data are not very convincing as they could be affected by an outlier effect, especially if derived from a single patient. This raises serious concerns and a more detailed analysis of the presented data is needed for a better-funded conclusion about this interesting topic. 
Please find my minor and major comments below.
Major comments
-	While the CT score changes significantly correlate with CRP and Gaffky scores (figure 2), the data are only derived from a handful of patients and could be largely driven by a single outlier. Each panel in figure 2 has a data point in the upper-right corner that stands alone and separated from the rest of the patients. Do these data point belong to the same single patient or multiple individuals? If these data originate from the same patient, they could easily bias the presented data and question whether there is a real correlation between the CT score change (per month or over the entire period of time) and the microbiological/immunological measures.
-	Would the data remain significantly correlated in each of the four panels in figure 2 if the upper/right outliers are omitted from the analysis?

Minor comments
-	Fonts in table 2 are very small and should be increased for better visibility
-	Which statistical method was used to establish the significance of the regression analysis?
